# Genomic Mutation Landscape of Primary Breast Lymphoma: Next-Generation Sequencing Analysis

**DOI:** 10.1155/2022/6441139

**Published:** 2022-07-29

**Authors:** Wenqi Zhang, Chen Huang, Jingjing Liu, Lili Wu, Huichao Zhang, Xiaolin Wu, Lianjing Wang, Weijing Li, Wei Liu, Lihong Liu

**Affiliations:** ^1^Department of Hematology, The Fourth Hospital of Hebei Medical University, Shijiazhuang, 050000 Hebei, China; ^2^Hebei Provincial Key Laboratory of Tumor Microenvironment and Drug Resistance, Shijiazhuang 050011, China; ^3^Research Center, The Fourth Hospital of Hebei Medical University, Shijiazhuang, 050000 Hebei, China; ^4^Clinical Laboratory, The Fourth Hospital of Hebei Medical University, Shijiazhuang, 050000 Hebei, China

## Abstract

Primary breast lymphoma (PBL) is a rare subtype of non-Hodgkin's lymphoma (NHL) with rapid progression and high risk of central nervous system metastasis. We have investigated 40 PBL patients retrospectively, and 16 of them were sequenced by a target panel of 112 genes related with lymphoma. Next-generation sequencing (NGS) identified 203 mutations spanning 35 genes and revealed seven potential protein-changing genes (PIM1, MYD88, DTX1, CD79B, KMT2D, TNFAIP3, and ITPKB) with high frequency, referring crucial roles in lymphomagenesis. Our result suggested that PIM1 mutation is correlated with the age and pathological type of PBL patients. Gene TNFAIP3 and KMT2D mutation is only related to the pathological type and primary site, respectively. These high-mutant genes detected in PBL indicated a tendency to shorten overall survival (OS) and progression-free survival (PFS), which may lead to poor prognosis. Furthermore, the nuclear factor kappa-B (NF-*κ*B) pathway and related regulatory factors are essential for the development of targeted therapy as well.

## 1. Introduction

Primary breast lymphoma (PBL) is defined as an extranodal lymphoma which presents as primary lesion within the breast without involvement of extramammary sites. It possesses an extremely rare incidence with rapid progression, poor prognosis, and high risk of central nervous system (CNS) relapse. The most common symptom is painless progressive breast mass with or without ipsilateral axillary fossa lymphadenectasis and usually involves the right breast for unknown reason. Histopathologic subtypes of PBL are variable including mucosa-associated lymphoid tissue (MALT) lymphoma, natural killer (NK)/T cell lymphoma, and T lymphocyte lymphoma, but diffuse large B-cell lymphoma (DLBCL) occurs in most cases [1–5]. At present, the most commonly applied chemotherapy regimen is rituximab combined with CHOP (cyclophosphamide, vincristine, doxorubicin, and prednisone) or CHOP-like treatment without any standard regimen. Prior studies suggest that the 5-year overall survival (OS) rate of PBL may reach 87% in the rituximab era.

However, due to the rarity of PBL, its predictive mutational signature and genetic features remain poorly understood. Therefore, efforts are required to explore better prognosis prediction and improve the cure rate of this invasive disease in today's precision medicine era. The advancement of gene profiling technologies such as next-generation sequence (NGS) has significantly contributed to prognosis and therapy guidance for lymphomas. For example, CD47 has become a novel therapeutic strategy for DLBCL according to previous works [6]. Recently, the role of the signal transduction pathway in tumor received particular interest. It is discovered that no matter what the pathological type of lymphoma is, the genetic changes in the nuclear factor kappa-B (NF-*κ*B) pathway are the most consequential and closely associate with prognosis by enrichment analysis of mutant genes. Here, we conducted the next-generation sequencer with high accuracy for screening genetic mutations through pathological tissues of 16 cases. Aimed to lay foundations for accurate diagnosis and applicable treatment methods, we have analyzed the correlation between gene mutation spectrum and clinical characteristics of PBL.

## 2. Experimental Section

### 2.1. Patients

Our investigation cohort collected 40 patients who were diagnosed with primary breast lymphoma and treated in the Fourth Hospital of Hebei Medical University from June 2009 to October 2019. Their diagnosis and histopathologic subtypes were determined on tumor tissues through standard immunohistochemistry procedures. Meanwhile, pathology results were observed and analyzed independently by more than 2 experienced pathologists. The study was approved by the ethics committee of the fourth hospital of Hebei Medical University. Because of the rare incidence of this disease, we are trying to evaluate as much patients that we selected as possible. The baseline clinical characteristics of 40 patients were summarized in Table 1. Detailed description will focus on 16 patients who underwent NGS, including treatment, surgery approaches, and outcomes (Table 2). The basis of screening for sequencing was mainly dependent on patients' will. Collectively, 16 patients were female with a median age of 59 years (range: 28–71 years). There were 7 patients aged ≥60 years (43.75%) and 9 patients aged <60 years (56.25%). Regarding the international prognostic index (IPI), 9 patients (56.25%) had a low-risk (0–1) score, 4 patients (25%) had a medium-risk (2–3) IPI score, and 3 patients (18.75%) had a high-risk (4–5) score. Only 2 of them (12.5%) presented with B symptoms, 4 patients (25%) showed increasing lactate dehydrogenase (LDH) (>250 *μ*/l), and 3 patients (18.75%) had an elevated *β* 2 microglobulin level (>2.7 *μ*g/ml). The pathological types were DLBCL in 14 cases (87.5%) and MALT lymphoma in 2 cases (12.5%) and 8 patients with Ki-67 index ≥ 70% among them (54.69%). All patients were receiving surgical treatment in the breast surgery department before chemotherapy. As for surgical approaches, modified radical mastectomy was conducted in 3 (18.75%) patients, total breast resection in 3 (18.75%) patients, and partial breast resection in 4 (25%) patients. Tumor resection was performed in 4 (25%) patients, and in 2 patients (12.5%), only tumor biopsy was performed. PBL is an aggressive subtype of non-Hodgkin lymphoma (NHL) with high risk of CNS metastasis. There were only 3 patients that presented with CNS metastasis and 10 patients with rapid progression.

A total of 15 patients underwent treatment, 12 (75%) only accepted chemotherapy or immunotherapy, 3 (21.43%) received radiotherapy combined with chemotherapy, and 3 (21.43%) combined intrathecal injection for CNS prevention. Just one patient received a novel option involving autologous stem cell transplantation (ASCT) and chimeric antigen receptor T cell therapy (CART) with radiotherapy plus chemotherapy.

### 2.2. Tissue DNA and NGS Library Preparation

The lymphoma plasma panel (Burning Rock Biotech, Guangzhou, People's Republic of China) is targeting 112 genes and spanning 314 K of human genomic regions, which strongly associated with the pathogenesis of lymphoma as well as its targeted therapy. Genomic DNA from 16 PBL patients was extracted from formalin-fixed paraffin-embedded (FFPE) samples (QIAamp DNA FFPE tissue kit; QIAGEN, Valencia, CA). The concentration of DNA was evaluated with the Qubit dsDNA assay (Life Technologies, Carlsbad, California) to ensure that genomic DNA was greater than 40 ng. Fragments of 200 to 400 bp sizes were selected with beads (Agencourt AMPure XP kit; Beckman Coulter, Brea, CA). Then, hybridization with the capture probe baits, hybrid selection with magnetic beads, and PCR amplification were subsequently conducted. The quality and size range assessment was performed by a high-sensitivity DNA assay. Pair-end read sequencing was then operated on the MiSeq Aystem (Illumina, San Diego, CA).

### 2.3. Bioinformatics Analysis

Sequencing data were mapped to the human genome (hg19) using BWA aligner 0.7.10. Local alignment optimization and variant calling was performed by GATK v3.2-2 (Broad Institute, Cambridge, Massachusetts) and VarScan (Genome Institute, Washington University, Washington, DC). The aimed average sequencing depth for all targeted regions was 2000x, and loci with a depth of less than 100 were filtered out with the VarScan filter pipeline. DNA translocation analysis was carried out with Tophat2 (Center for Computational Biology, Johns Hopkins University, and the Genome Sciences Department, University of Washington) and FACTERA1.4.3. The insertion size distribution and library complexity were calculated to evaluate the level of DNA degradation across all tissue samples. In order to avoid false-positive mutation calls due to DNA damage, we used different mutation thresholds on samples with different DNA quality. Single-number variations (SNVs) and indel identification were evaluated with the dbNSFP (v30a), COSMIC (v69), and dbSNP (snp138) databases. Copy number variation (CNV) analysis was performed by normalizing and read counts from each target region. CNV on the gene level was assessed by the *z*-test. Single-nucleotide polymorphisms (SNPs) were defined as variants with a global minor allele frequency greater than 1.0% in the 1000 Genome Project, which has not been discussed.

### 2.4. Follow-Up & Statistical Analysis

Our follow-up methods are using telephone investigation as well as inpatient or outpatient evaluation. OS was calculated from diagnosis to death as any cause or final follow-up. Progression free survival (PFS) was defined from the beginning of treatment to the date disease progression, recurrence, death, or final follow-up. The end point is on February 2020, and the median follow-up time is 23 months. The Kaplan-Meier method was used for univariate analysis, and the log rank test for factor comparison and survival analysis were performed according both of them. The Cox risk regression model was built for multivariate analysis of variables significantly affecting OS in univariate analysis. All statistical identifications were two-tailed tests, and *P* < 0.05 was considered statistically significant. The IBM SPSS 21.0 (Chicago, IL, USA, 2012) and software R, version 4.1.2 (http://www.R-project.org), were applied for data analyses.

## 3. Result

### 3.1. Survival Data

**Figure 1:**
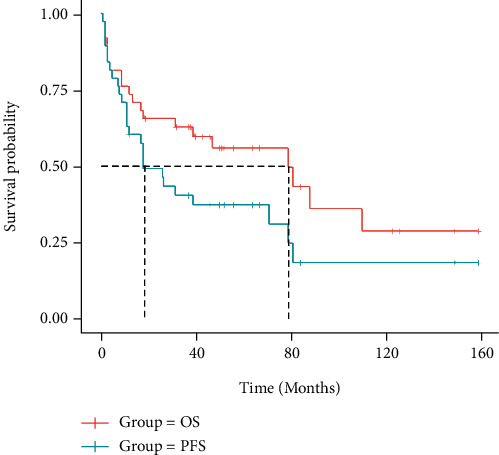
Survival curves of overall survival (OS) and progression-free survival (PFS) in the cohort of 40 patients.

Follow-up of 40 patients was complete through November 2021, 22 patients (55%) were still alive, 16 patients (40%) died, and 2 patients (5%) were lost at the end point. In our group, the median overall survival (OS) was 79 months, while the median progression free survival (PFS) was 39 months. (Figure 1) The 1-year, 3-year, and 5-year OS rates of 38 people were 72.97%, 59.46%, and 27.03%, respectively, and the 1-year, 3-year, and 5-year PFS rates were 64.86%, 37.84%, and 21.62%, respectively. Patients without marrow infiltration received longer OS compared to those involving marrow infiltration (median OS, 88 months vs 18 months, log rank = 4.923, *P* = 0.0265). Furthermore, patients in stage IV developed poorer OS in comparison to patients in stages I & II, which was diagnosed by Ann Arbor staging (median OS, 18 months vs 110 months, log rank = 4.843, *P* = 0.0278).

No difference was observed in both OS and PFS among the following clinical variables when analyzed individually: age, presence of B symptoms, IPI risk group, elevated LDH, and *β* 2 microglobulin level (*P* > 0.05). There was no significant difference in statistics but inferred a trend of shorter OS and PFS, which possibly led to inferior prognosis. Patients received different treatment regimens like chemotherapy combined with radiotherapy, applying rituximab with CHOP or CHOP like immune-chemotherapy, or accepting intrathecal injection to prevent central infiltration, etc. Different regimen presents no significant impact on further prognosis and survival.(*P* > 0.05).

### 3.2. Correlation of Oncogenic Alterations and Clinical Features

**Figure 2:**
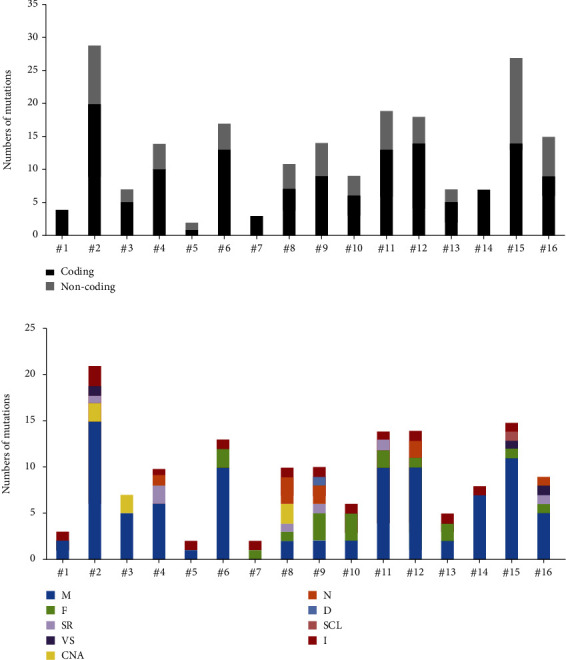
The mutation number of 16 PBL patients identified with NGS. (a) Total number of mutations in coding and noncoding regions; (b) distribution of missense (M), frameshift (F), nonsense (N), infusion (I) mutation, shear region (SR) mutation, variable shear region (VS) mutation, start codon loss (SCL), and copy number amplification (CNA) in coding regions of targeted genes.

**Figure 3:**
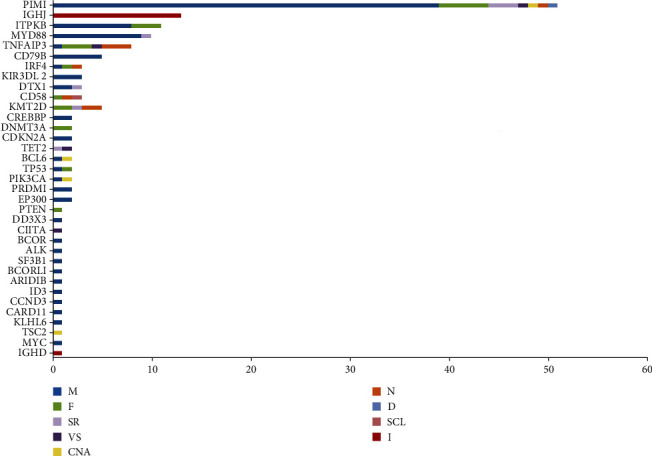
Mutation numbers and distributions in the coding regions of 35 targeted genes from NGS.

**Figure 4:**
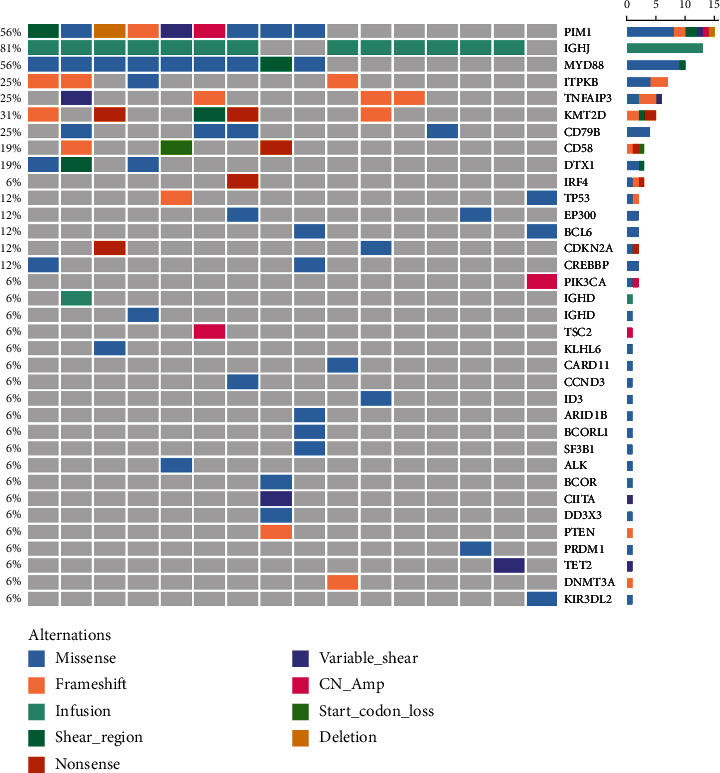
The oncoPrint heat map indicates mutational spectrum of 35 oncogenes and distribution of genetic alterations in coding regions analyzed by NGS from 16 PBL patients. Genetic alteration types are labeled in color legend, particular genes in rows, and patient samples in columns. Mutation rates for each gene are demonstrated on the left of the graph.

To profile the molecular mutation landscape and pathogenesis of PBL, our NGS identification covers 112 hotspot exon regions and 11 intron regions from 16 patients in this cohort. Their clinical characteristics and mutation spectrum were demonstrated in Table 3 and Figure 2, respectively. The top high-frequency mutant genes included PIM1 (68.75%), MYD88 (56.25%), DTX1 (31.25%), CD79B (31.25%), KMT2D (31.25%), TNFAIP3 (25%), and ITPKB (25%). We further analyzed the correlation between oncogenic alterations of 203 mutations from 35 genes and other clinical features. Most individuals (81.25%) had immunoglobulin heavy locus (IGH) infusion, except #3, #14, and #16. Infusion mutations contain two IGHJ2, two IGHJ3, six IGHJ4, five IGHJ6, and one IGHD4-4 rearrangements, which always indicates the pathological state of the B cell cloning process. Variation among those genes had missense mutation, frameshift mutation, nonsense mutation, deletion mutation, infusion mutation, shear region mutation, variable shear region mutation, start codon loss, intron variation, and copy number amplification (Figures 3 and 4). In 16 PBL patients, we evaluated a total of 140 mutations in coding regions (median per patient: 8, range: 1–20), which referred to potential protein changing, and 63 mutations in noncoding regions (median per patient: 4, range: 0–13). Just two patients had over 25 mutations: #2 and #15. Missense mutation occurred in most cases.

As one of the most highly mutant gene, PIM1 revealed correlations between the age (*P* = 0.015) and pathological type (*P* = 0.024) of PBL patients. The alternations occurred in 10 patients involving 30 synonymous mutations and 67 nonsynonymous mutations with median OS of 21 months. The TNFAIP3 gene mutation contains 3 frameshift mutations, 3 nonsense mutations, 1 missense mutation, and 1 variable shear mutation in 4 patients, which is only associated with the pathological type of PBL (*P* = 0.006). The median OS of TNFAIP3 mutant and nonmutant patients were 21 and 23 months, respectively. Five patients presented KMT2D gene mutation with 2 nonsense mutations, 2 frameshift mutations, and 1 shear region mutation. It showed the relationship with the primary site (*P* = 0.004). Additionally, we pay more attention to MYD88 and CD79B mutation in PBL. In our group, there are 9 patients with MYD88 mutation and 5 patients with CD79B mutation and 5 of them have double mutation. These mutations did not make any significant difference on the survival and other clinical features (including the primary site, Ann Arbor stage, IPI score, LDH and *β* 2 micro-globulin levels, bone marrow infiltration, B symptoms, and Ki-67); there was no significant correlation (*P* > 0.05).

## 4. Discussion

In recent years, a lot work focusing on the signal transduction pathway and gene alternation in tumor researches captured much attention. With the advancements of NGS technology, the molecular landscape of PBL could be profiled in order to explore the standard treatment scheme and realize individualized precision treatment for our patients. Due to the low incidence rate, its clinical characteristic and genetic features required further investigation. We detected biopsy specimens of 16 PBL patients by NGS technology, and biological effects of related genes, pathogenesis, and prognosis were also analyzed. There were 7 high-mutation frequent genes among 112 gene panels including PIM1, MYD88, DTX1, CD79B, KMT2D, TNFAIP3, and ITPKB.

PIM1 is a serine/threonine kinase acting as the most common mutant gene in PBL involving a series of biological functions like survival, proliferation, and differentiation. It has universally acknowledged the critical role of PIM1 in the occurrence and development of hematological malignancies and identified as the target of abnormal somatic hypermutation in DLBCL [7–9]. PIM family kinases were activated by JAK-STAT signaling pathways and downstream of NF-*κ*B transcription factors, inducing and regulating protein activity to promote tumorigenesis. Plenty of studies claimed that the PIM kinase inhibitor has become a promising candidate of highly specific and selective drugs with superior toxicity characteristics [10, 11].

Genes MYD88 L265P and CD79B were essential in PBL lymphomagenesis and were frequently detected in PBL. There were totally 9 patients that harbored MYD88 L265P mutation; it was functional gain-driven mutation and located in the MYD88 Toll-like/interleukin- (IL-) 1 receptor domain, which is related with unfavourable prognosis among aggressive lymphoma. L265P mutants promote cell survival by spontaneously assembling protein complexes containing interleukin-1 receptor-associated kinase 1 (IRAK1) and interleukin-1 receptor-associated kinase 4 (IRAK4) [12]. It may lead the activation of IRAK4, NF-*κ*B signaling, JAK kinase in signal transducer and activator of transcription 3 (STAT3), IRAK phosphorylation, and secretion of IL-6, IL-10, and interferon *β* (IFN*β*). Zhang et al. hold the view that downregulation of IRAK4 and NF-*κ*B may cause tumor matrix disorganization through investigation. Consequently, the signal complex coordinated by MYD88 and L265P is an appealing target and the IRAK4 kinase inhibitor is also expected to become a therapeutic approach for malignancies with MYD88 mutation. The CD79B gene, encoding a subunit of the B-cell antigen receptor, contains the domain of immune receptor tyrosine activation motif (ITAM) which plays a critical role in signal transduction from the B-cell receptor (BCR). The presence of CD79B mutations has always been recognized in the first tyrosine (Y196) of ITAM [13, 14]. Previous findings described the upregulation of BCR on the cell surface resulting in NF-*κ*B signaling activation via tyrosine protein kinase (LYN), tyrosine kinase in the spleen (SYK), and the tyrosine kinase of Bruton (BTK), which inhibits B cell apoptosis and promotes proliferation in lymphoma cells [15, 16]. In B cells, NF-*κ*B signaling was initially transferred by CD79A (Ig-*α*) and CD79B (Ig-*β*). Meanwhile, the phosphorylated ITAM mediated with SYK activation triggered signal cascade reaction involving BTK, PLC*γ*, and PKC*β*. Then, the “CBM complex” was formed and activated direct phosphorylation of I*κ*-B kinase (IKK) to start transduction [14]. Genes MYD88, PIM1, and CD79B were connected and interacted with each other according to the NF-*κ*B pathway as the primary target signal node. TNFAIP3, also known as A20, represents a tumor suppressor gene in lymphomas that synergistically attenuates NF-*κ*B signaling induced by tumor necrosis factor (TNF) and TLR signal transduction. Alternations including deletion, promoter methylation, point mutation, and frameshift mutation lead to inactivation of RIP protein ubiquitination mediated by TNF [17, 18].

Meanwhile, epigenetic gene like KMT2D has stimulation on the methylation of H3K4 and modulates genes related with B cell differentiation containing CD40, JAK-STAT, and TLR. Previously published studies pointed out that the deficiency and mutations in KMT2D could hinder B cell differentiation and delay germinal central degeneration, thus resulting in rapid progression and high mortality with lymphoma [19, 20]. Numerous works confirmed that calcium reaction was a key factor during cancer invasion and metastasis. ITPKB becomes a target during the process by encoding isoenzymes of inositol 1,4,5–triphosphate 3-kinase (Ins(1,4,5) p3) in B cells.

The NF-*κ*B pathway modulated cell proliferation and angiogenesis by transcriptional regulation and inhibiting apoptotic genes resulting in tumorigenesis. The signaling could be stimulated according to activation mutations in CD79B and MYD88 and inactivation mutations in TNFAIP3. Shorter survival was observed in patients mutated with PIM1, MYD88, CD79B, TNFAIP3, DTX1, KMT2D, and ITPKB, consisting with other literatures, but there was no statistically significant correlation between alternations and prognosis. However, the statistical results of such small cohort may not be representative. Considering the rare incidence of PBL and limited sample size, further study could be verified in a large cohort trail.

## 5. Conclusion

In summary, the NGS analysis for 16 PBL patients among 35 hotspot genes provides an implication that PIM1, MYD88, DTX1, CD79B, KMT2D, TNFAIP, and ITPKB are possibly consistent with the trend for shorter survival and poor prognosis as high-frequency mutant genes. Nevertheless, we found that patients diagnosed with marrow infiltration and stage IV are correlated with inferior overall survival. Other clinical characteristics and therapy approaches make no significant differences on survival status.

## Figures and Tables

**Table 1:** Baseline clinical characteristics of the total 40 primary breast lymphoma (PBL) patients.

Characteristics	*n* = 40	Percentage (%)
*Gender*		
Female	38	95
Male	2	5
*Age*		
⩾60	13	32.5
<60	27	67.5
*Ann Arbor stage*		
I–II	19	47.5
III–IV	21	52.5
*Ki-67*		
⩾70	28	70
<70	12	30
*Bone marrow infiltration*		
Absent	32	80
Present	8	20
*B symptom*		
Absent	32	80
Present	8	20
*IPI*		
0–2	33	82.5
3–5	7	17.5
*LDH*		
Normal	24	60
Increase (>250)	16	40
*β2-MG*		
Normal	33	82.5
Increase (>250)	7	17.5
*Treatment*		
RT + CT	8	20
RT	1	2.5
CT	26	65
No treatment	5	12.5
*Pathologic types*		
DLBCL	32	80
MALT	3	7.5
NK/T	1	2.5
T cell	1	2.5
B-LBL	1	2.5
PBL	1	2.5
*Original site*		
Left breast	19	47.5
Right breast	20	50
Bilateral breast	1	2.5

**Table 2:** Clinical characteristics of 16 patients among the total 40 PBL patients undergoing next-generation sequence (NGS) analysis.

No.	Gender	Age	IPI	LDH	Surgical approach	Primary size (cm × cm)	Treatment	Extranodal metastasis	Response vital	Status
#1	F	49	1	Normal	MRM	7.0 x 6.1	C + R	BM	SD	Dead
#2	F	59	2	Normal	TBR	5.0 x 3.0	None	No	NA	Alive
#3	F	46	0	Normal	PBR	7.0 x 5.0	C + R + ASCT	No	CR	Alive
#4	F	59	0	Normal	MRM	3.5 x 3.0	C + IT	No	CR	Alive
#5	F	46	0	Normal	TR	3.0 x 2.0	C + R	Bone	Cru	Alive
#6	F	67	1	Normal	MRM	4.0 x 3.5	C + IT	No	CR	Alive
#7	F	54	0	Normal	PBR	3.0 x 3.0	R	No	CR	Alive
#8	F	65	2	Elevated	PBR	4.0 x 3.0	C	Breast & face	PR	Alive
#9	F	71	1	Normal	TR	1.5 x 1.0	C	No	CR	Alive
#10	F	64	4	Elevated	TR	3.0 x 3.0	C + IT	Bone, breast, kidney	Cru	Alive
#11	F	71	4	Normal	PBR	2.0 x 2.0	C	Bone	NA	Dead
#12	F	60	2	Normal	TB	4.0 x 3.0	C	BM	Cru	Alive
#13	F	51	2	Elevated	TBR	2.0 x 2.0	C	No	PR	Dead
#14	F	71	4	Elevated	TB	4.0 x 3.0	C	No	CR	Dead
#15	F	28	0	Normal	TBR	1.5 x 1.5	C + R + ASCT+CART	No	CR	Dead
#16	F	40	0	Normal	TR	3.0 x 2.5	C	No	Cru	Dead

MRM: modified radical mastectomy; TBR: total breast resection; PBR: partial breast resection; TR: tumor resection; TB: tumor biopsy; C: chemotherapy; R: radiotherapy; ASCT: autologous stem cell transplantation; CART: chimeric antigen receptor T cell therapy.

**Table 3:** Identification of 35 genes affected by potential protein-changing mutations (missense, frameshift, nonsense, deletion, infusion, shear region mutation, variable shear region mutation, start codon loss, and copy number amplification) in 16 PBL patients. The high-frequency mutated genes including PIM1, IGHJ, MYD88, DTX1, CD79B, KMT2D, TNFAIP3, and ITPKB.

Gene	Numbers of patients	Numbers of mutation
*PIMI*	10	51
*IGHJ*	13	13
*ITPKB*	4	11
*MYD88*	9	10
*TNFAIP3*	4	8
*KMT2D*	5	5
*CD79B*	5	5
*CD58*	3	3
*DTX1*	3	3
*KIR3DL2*	1	3
*IRF4*	1	3
*EP300*	2	2
*PRDMI*	2	2
*PIK3CA*	1	2
*TP53*	2	2
*BCL6*	2	2
*TET2*	2	2
*CDKN2A*	2	2
*DNMT3A*	1	2
*CREBBP*	2	2
*IGHD*	1	1
*MYC*	1	1
*TSC2*	1	1
*KLHL6*	1	1
*CARD11*	1	1
*CCND3*	1	1
*ID3*	1	1
*ARIDIB*	1	1
*BCORLI*	1	1
*SF3B1*	1	1
*ALK*	1	1
*BCOR*	1	1
*CIITA*	1	1
*DD3X3*	1	1
*PTEN*	1	1

## Data Availability

Our NGS results were processed by Burning Rock Biotech. We appreciated all the patients, families, and investigators that participated in our research.
